# *Crataegus Aronia* protects and reverses vascular inflammation in a high fat diet rat model by an antioxidant mechanism and modulating serum levels of oxidized low-density lipoprotein

**DOI:** 10.1080/13880209.2018.1564930

**Published:** 2019-01-31

**Authors:** Abdullah S. Shatoor, Suliman Al Humayed, Mahmoud A. Alkhateeb, Khalid A. Shatoor, Hussain Aldera, Mohammed Alassiri, Ali A. Shati

**Affiliations:** a Department of Medicine, Cardiology Section, College of Medicine, King Khalid University (KKU), Abha, Saudi Arabia;; b Department of Basic Medical Sciences, College of Medicine, King Saud bin Abdulaziz University for Health Sciences (KSAU-HS), Riyadh, Saudi Arabia;; c An intern, College of Medicine, King Khalid University (KKU), Abha, Saudi Arabia;; d King Abdullah International Medical Research center (KAIMRC), Riyadh, Saudi Arabia;; e Department of Biology College of Science, College of Medicine, King Khalid University (KKU), Abha, Saudi Arabia

**Keywords:** Hyperlipidemia, atherosclerosis, aorta, oxidative stress

## Abstract

**Context:***Crataegus aronia* (Willd.) Bosc (Rosaceae) (syn. *Azarolus* L) is traditionally used to treat cardiovascular disorders.

**Objectives:** To investigate *C. aronia* protection against a high-fat diet (HFD)-induced vascular inflammation in rats.

**Materials and methods:** Wistar Male rats (180–220 g) were divided (*n* = 10/group) as control fed a standard diet (STD), STD + *C. aronia* (200 mg/kg, orally), HFD, HFD + *C. aronia* and HFD post-treated with *C. aronia*. Simvastatin (20 mg/kg) was co- or post-administered as a positive control drug. HFD was given for 8 weeks, and all other treatments were administered for 4 weeks.

**Results:** Most significantly, co-administration of *C. aronia* to HFD-fed rats reduced the thickness of aorta tunica media (90 ± 5 vs. 160 ± 11.3 µm) and adventitia (54.3 ± 3.8 vs. 93.6 ± 9.4 µm). It also lowered protein levels of TNF-α (0.51 ± 0.15 and 0.15 ± 0.16 vs. 0.1 ± 0.09%) and IL-6 (0.52 ± 0.19 vs. 1.0 ± 0.2%) in their aorta or serum (5.9 ± 0.91 vs. 12.98 ± 1.3 ng/mL and 78.1 ± 6.7 vs. 439 ± 78 pg/mL, respectively). It also lowered all serum lipids and increased aorta levels of GSH levels (70.4 ± 4.0 vs. 40.7 µM) and activity of SOD (5.7 ± 0.7 vs. 2.9 ± 0.6 U/mg) and decreased serum levels of ox-LDL-c (566.7 ± 46 vs. 1817 ± 147 ng/mL). Such effects were more profound than all other treatments.

**Conclusions:***C. aronia* inhibits the HFD-induced vascular inflammation and its use in clinical trials is recommended.

## Introduction

While the aetiology of hyperlipidemia is known to be either primary (genetically determined) or secondary (acquired), the consumption of high-fat diets (HFD) in most areas of the world is considered the most common secondary predisposing factors for hyperlipidemia (Freire et al. 2005; Moreno and Rodríguez 2007). Hyperlipidemia is the major contributor to the development of atherosclerosis worldwide and its complications where vascular, metabolic and inflammatory components are involved in its pathogenesis (Libby 2006; Besler et al. 2012).

Hyperlipidemia-induced atherosclerosis is mainly initiated by vascular inflammation (Libby 2006). Accumulation of low-density lipoproteins (LDL) in the arterial wall and their oxidation by reactive oxygen species (ROS), which consequently forms and accumulates high levels of oxidized LDL (ox-LDL), triggers a complex series of biochemical and inflammatory reactions (Tavori et al. 2009; Anogeianaki et al. 2011). These inflammatory reactions are mediated by complex interactions between the circulating leukocytes and vascular cells [e.g., endothelial cells (ECs) and smooth muscle cells (SMCs)] (Galkina and Ley 2007). Vascular cells regulate the inflammatory process through the release of specific inflammatory cytokines, chemokines and growth factors and upregulate the expression of specific cell adhesion molecules (CAMs) including intercellular adhesion molecule-1 (ICAM-1) and vascular cell adhesion molecule-1 (VCAM-1), in response to pro-inflammatory cytokines, such as tumor necrosis factor-α (TNF-α) and interleukin-6 (IL-6) (Galkina and Ley 2007).

Investigating the vascular consequences of chronic hyperlipidemia, the pathologic mechanisms and the beneficial effects of ameliorative therapies have received considerable attention in recent years (Huijgen et al. 2008). Pharmaceutical therapies have been shown to influence the vascular inflammation through reduction of cholesterol level and inflammatory markers, thus stabilizing an existing plaque and thereby reducing the risk of acute vascular events (Chu et al. 2003). Statins are potent hypolipidemic agents which improve the outcome of ischemic heart disease irrespective of its hypolipidemic effect. These favourable effects of statins are believed to be partly due to its antioxidant and anti-inflammatory actions (Bondar et al. 2012). However, the use of statins is limited by their side effects such as myositis, hepatitis, and more recently recognized complication such as induction of diabetes (Escobar et al. 2008). Hence, a search of new, safe drug that may alter the process of atherosclerosis with little or no side effects is warranted.

*Crataegus aronia* (Willd.) Bosc (Rosaceae) (syn. *Azarolus* L), predominantly found in the mountains of the Mediterranean basin, is commonly used in the Arabic traditional medicine to treat cardiovascular diseases as well as cancer, diabetes, hyperlipidemia and sexual weakness (Ljubuncic et al. 2005). In our labs, we have shown that *C. aronia* is a well-tolerated plant (LD_50_ is up to 2000 mg/kg) and have several beneficial effects on the cardiovascular system including an antiplatelet, hypolipidemic, inotropic, heart rate lowering and antioxidant effects (Shatoor 2011, 2012; Shatoor 2013; Humayed 2017).

However, the protective effect of *C. aronia* against HFD-induced vascular inflammation was not investigated before. Hence, in this study, we examined a sub-chronic administration of *C. aronia* on ameliorating hyperlipidemia-induced aortic inflammation and thickened aortic media in a rat model fed HFD. We also compared these effects with simvastatin and examined some mechanisms by which *C. aronia* may act.

## Materials and methods

### Preparation of the extract

This study was carried out at the College of Medicine of King Khalid University (KKU), Abha, Saudi Arabia. Aerial *C. aronia* including stems, flowers and leaves (with no roots) were purchased in January 2017, from a local licensed herbal plant supplier market (Kabatilo Natural products store) in Jordan (Middle-east), where the collection records indicated that the plant was dried and preserved naturally for only 1 month. The plant was identified by Hesham Solaiman, a professor in the Department of Pharmacognosy at the College of Pharmacy, King Khalid University based on an available voucher specimen. The aqueous extract was prepared at the pharmacognosy laboratories in the College of Pharmacy in accordance with our previously published method (Shatoor 2011, 2013; Shatoor et al. 2012). In brief, the dried plant material was ground to a powder and extracted by maceration using distilled water (1 kg/1 L, *w*/*v*) for 3 days at 37 °C. The extract was filtered and evaporated under reduced pressure in a rotary evaporator. The resulting residue (40 g) was stored at 4 °C. The residue was reconstituted in distilled water to a final concentration of 200 mg/mL and used in this study.

### Experimental animals

A total of 70 adult male Wistar rats, 6 weeks old and weighing 180–220 g were obtained from the rat breeding colony at the animal house of the College of Medicine at KKU, Saudi Arabia. The rats were housed 10 per cage. The rats of all groups were preconditioned for 1 week before the beginning of the treatment protocol. During this period, the rats received a normal rodent’s chow diet and water *ad libitum* and were kept in a room where the temperature was maintained at 22 ± 2 °C, relative humidity at 55 ± 10% and a 12 h light/dark cycle. All experiments were conducted under the protocol approved by the ethical committee, KKU, Saudi Arabia (REC # 2013-03-06). All procedures involving rats were performed in strict compliance with relevant laws, the Animal Welfare Act, Public Health Services Policy, and guidelines established by the National Institute of Health Guide for the Care and Use of Laboratory Animals.

### Experimental design

**Figure 1.**
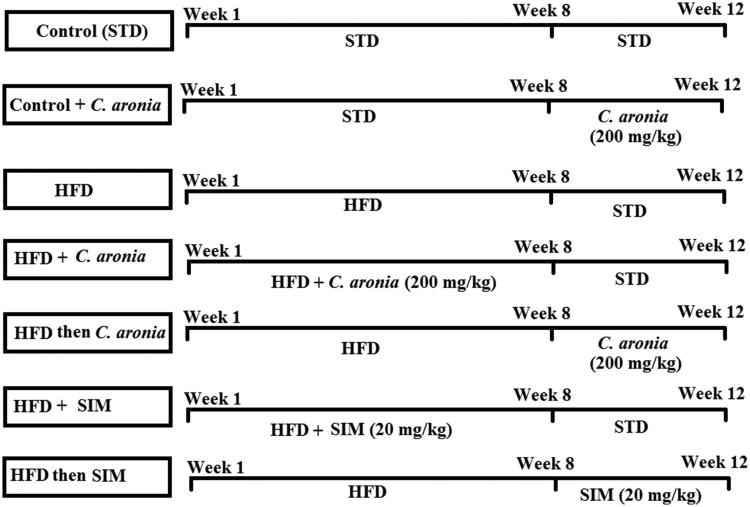
Schematic diagram for the experimental groups and experimental procedure of the study.

After 1 week of adaptation, the rats were divided into seven groups (10 rats each) as (1) A control group: fed a standard diet (STD) (12% of calories as fat) for 12 weeks; (2) control + *C. aronia*-treated rats: fed STD for 8 weeks and then received *C. aronia* (200 mg/kg/day) on the last 4 weeks; (3) HFD-induced rats: fed HFD (40% of calories as fat) for 8 weeks and then continued on STD for the next 4 weeks; (4) HFD + *C. aronia*-treated rats: fed HFD and received a concomitant dose of *C. aronia* (200 mg/kg/day) for 8 weeks and then continued on STD for the next 4 weeks; (5) HFD then *C. aronia*-treated rats (HFD then *C. aronia)*: fed HFD for 8 weeks and then post-treated with *C. aronia* (200 mg/kg/day) for the next 4 weeks; (6) HFD + simvastatin (SIM)-treated rats (HFD + SIM): fed HFD and received a concomitant dose of SIM (20 mg/kg/day), as a positive control drug for 8 weeks and then continued on STD for next 4 weeks; and (7) HFD then SIM-treated rats (HFD then SIM): fed HFD for 8 weeks and then post-treated with simvastatin (20 mg/kg/day) for next 4 weeks. A summary of the experimental procedure is shown in Figure 1.

The ingredients and chemical composition of STD and HFD diet have been described previously by others (Tuzcu et al. 2011) and are shown in Table 1. In our preliminary studies, the peak of change in serum levels of ox-LDL, serum triglycerides (TGs), and cholesterol (CHOL), low-density lipoprotein-cholesterol (LDL-c), high-density lipoprotein cholesterol (HDL-c) and very low-density lipoprotein-cholesterol (VLDL-c) as well ICAM-1 and VCAM-1 were seen between weeks 8 and 12. Since there are no significant differences in all of the tested biochemical levels seen at 8 weeks as compared to their levels measured by the end of week 12 (data not shown), we decided to give HFD for 8 weeks. All treatments were induced orally to rats. The selected doses of *C. aronia* and SIM used in this study were based on previous reports which showed their effective cardioprotective and hypolipidemic effects at these doses (Shatoor 2011, 2013; Shatoor et al. 2012; Humayed 2017). Both the STD and HFD were prepared weekly and along with the treatments were always stored at the 4 °C cold chamber.

**Table 1.** Ingredient and nutrient composition of the high-fat diet.

Ingredients (g/kg)	Standard Diet	High-Fat Diet (HFD)
Casein	200.0	200.0
Starch	615.0	145.0
Sucrose	000.0	150.0
Corn oil	080.0	000.0
Beef tallow	000.0	400.0
Cellulose	050.0	050.0
Vitamin-Mineral Premix	050.0	050.0
DL-Methionine	003.0	003.0
Choline chloride	002.0	002.0

### Serum and tissue collection

At the end of week 12, all rats were fasted overnight for 12 h and then were anesthetized with sodium pentobarbital (65 mg/kg, i.p.). Blood samples (3 mL) were collected directly from their hearts into plain tubes, settled for 30 min and then centrifuged at 5000 rpm for 5 min to collect sera which were used later to measure lipid profile and inflammatory marker levels. All animals were then killed ethically by cervical dislocation. Fresh abdominal aorta samples were collected on ice, washed with ice-cold phosphate buffer saline (PBS, pH 7.4) and were cut in cross sections into small pieces. Some samples of each aorta/rat were placed in 10% buffered formalin for 24 h and then used for histopathological staining, while other samples were frozen at −80 °C and used for biochemical and western blot analysis.

### Serum and tissue biochemistry

The concentrations of serum levels of CHOL, TGs, LDL-c, HDL-c and VLDL-c were measured using the corresponding commercial enzyme kits (Human Diagnostic Worldwide, Wiesbaden, Germany). Serum levels of ICAM-1 (Cat No. MBS267983), VCAM-1 (Cat No. MBS027532), high sensitive C-reactive protein (hsCRP, Cat. No. MBS008334), TNF-α (Cat. No. MBS267737) and IL-6 (Cat No. MBS355410) were measured using assay kits purchased from (MyBioSource, CA, USA). All measurements were performed in duplicate of 10 samples/group. All procedures followed the manufacturer’s instructions.

### Biochemical analysis in the aorta samples

Frozen aorta samples were homogenized in the ice-cold PBS (pH = 7.4), centrifuged at 10,000 rpm and the collected supernatants were stored at −80 °C. These frozen supernatants were used later for determination of levels reduced and oxidized glutathione (GSH and GSSG; cat. no. ab156681, Abcam, Cambridge, UK), malondialdehyde (MDA; cat. no. NWK-MDA01, Northwest Life Science Specialist (NWLSS), Toronto, Canada) and activity of Superoxide dismutase (SOD; cat. no. 706002, Cayman Chemical, Ann Arbor, MI). All procedures were done in accordance with the manufacturer’s instructions.

### Western blotting

Whole cell proteins were extracted from frozen aortas using RIPA buffer [150 mM sodium chloride 1.0% NP-40 or Triton X-100 0.5% sodium deoxycholate 0.1% SDS (sodium dodecyl sulfate) 50 mM Tris, pH 8.0] to which protease inhibitor cocktail was added (Cat. No. P8340, Sigma-Aldrich, St. Louis, MO). The protein concentrations were measured using a Bradford assay. Proteins were then separated on 10% SDS-PAGE gel and transferred to nitrocellulose membranes, blocked with skimmed milk and then incubated overnight at 4 °C with primary antibodies against IL-6 (Cat No. sc-28343, 21 kDa, 1:1000, Santa Cruz Biotechnology, TX, USA), TNF-α (Cat. No. sc-52746, 26 kDa, 1:1000, Santa Cruz Biotechnology), and β-actin (Cat. No. 4967, 45 KD, 1:2000, Cell Signaling Biotechnology, MA, USA). Then, all membranes were incubated with their corresponding horseradish peroxidase-conjugated antibodies overnight at 4 °C. Antigen-antibody reactions were then detected using a Pierce ECL kit (ThermoFisher, Piscataway, NJ) and bands intensities were measured using C-DiGit Blot Scanner (LI-COR Biosciences, Nebraska, USA) and analyzed using Image Studio DIGits software (LI-COR Biosciences, Nebraska, USA). The ratio of each protein is presented as relative protein expression to the reference protein, β-actin. Data are represented as mean ± SD of 6 samples/group.

### Histopathological evaluation

For light microscopy, the isolated aortic tissues were cleaned, dried, and then fixed in a buffer solution of 10% neutral buffered formalin. The fixed tissues were embedded in paraffin, cut at spam thickness and stained with hematoxylin and eosin (H&E) for general histopathological evaluation. Aorta thickness was analyzed with Image J free online software.

### Statistical analysis

Graphing and comparison between the groups were analyzed by one-way ANOVA test using GraphPad Prism (Version 6, Sydney, Australia) followed by Tukey’s *t*-test, and the data are expressed as mean ± SD. A value of *p* < 0.05 was considered to be statistically significant.

## Results

### Changes in the serum lipid parameters in all groups

**Figure 2.**
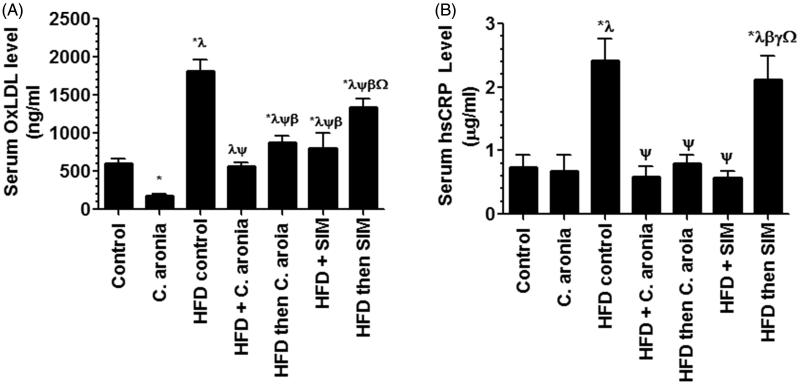
Levels of oxidized LDL-c (ox-LDL, A) and high sensitive C-reactive protein (CRP, B) in the sera of all experimental groups. Values are expressed as Mean ± SD for ten rats in each group and were considered significantly different at *p* < 0.05. *Significantly different when compared to control group. ^λ^Significantly different when compared to group Control + *C. aronia*. ^ψ^Significantly different when compared to group HFDI. ^β^Significantly different when compared to group HFD + *C. aronia*. ^γ^Significantly different when compared to group HFD then *C. aronia*. ^Ω^Significantly different when compared to HFD + SIM. HFD: high-fat diet; SIM: simvastatin.

Table 2 summarizes the changes in the lipid parameters in all groups. There is a significant decrease in levels of TGs, CHOL, LDL-c (Table 2) and ox-LDL (Figure 2(A)) with no alterations in levels of VLDL-c and HDL-c (Table 2) in the sera of control rats received the *C. aronia* as compared to control rats fed STD only. On the other hand, HFD-model rats or HFD rats post-treated with SIM showed significant elevations in serum levels of all lipid parameters including TGs, CHOL, LDL-c, VLDL-c (Table 2) and ox-LDL-c (Figure 2(A)) and showed significant decreases in serum levels of HDL-c as compared to the control group (Table 2). However, there were no significant differences in the levels of all lipid parameters between HFD and HFD then SIM-treated groups. HFD-fed rats received the concomitant doses of *C. aronia* or SIM or post-treated *C. aronia* showed significant decreases in the serum levels of TGs, CHOL, LDL-c, VLDL-c (Table 2) and ox-LDL-c (Figure 2(A)) with significant a parallel increases in serum levels of HDL-c, compared to HFD-fed rats (Table 2). However, the most significant effect is observed in HFD+ *C. aronia* treated rats as compared to all other groups (Table 2) and (Figure 2(A)).

**Table 2.** Levels of serum lipids in the control and all experimental groups.

	Control	Control + *C. aronia*	HFD	HFD + *C. aronia*	HFD then *C. aronia*	HFD + SIM	HFD then SIM
TG (mg/dl)	42 ± 6.1	34 ± 4.4^a^	271 ± 17.4^a^^,^^b^	89 ± 4.4^a^^,^^b^^,^^c^	114 ± 8.9^a^^,^^b^^,^^c^	84 ± 4.6^a^^,^^b^^,^^c^	129.3 ± 4.6^a^^,^^b^^,^^c^^,^^d^^,^^e^
CHOL (mg/dl)	63 ± 4.8	57 ± 4.7^a^	169 ± 3.66^a^^,^^b^	75 ± 1.8^b^^,^^c^	106 ± 3.2^a^^,^^b^^,^^c^^,^^d^	108 ± 2.9^a^^,^^b^^,^^c^^,^^d^	114.8 ± 4.2^a^^,^^b^^,^^c^^,^^d^
LDL-c (mg/dl)	31 ± 1.6	25 ± 1.7^a^	93 ± 4.5^a^^,^^b^	31 ± 0.7^c^	61 ± 1.9^a^^,^^b^^,^^c^^,^^d^	72 ± 2.7^a^^,^^b^^,^^c^^,^^d^^,^^f^	74.4 ± 3.3^a^^,^^b^^,^^c^^,^^d^^,^^f^
HDL-c (mg/dl)	24 ± 1.8	25 ± 1.8	15 ± 1.0^a^^,^^b^	27 ± 1.9^a^^,^^c^	19 ± 0.8^a^^,^^b^^,^^c^^,^^d^	17 ± 1.4^a^^,^^b^^,^^d^	16.1 ± 0.5^a^^,^^b^^,^^d^
VLDL-c (mg/dl)	8 ± 1.2	7 ± 0.9	55 ± 8.3^a^^,^^b^	17 ± 0.9^a^^,^^b^^,^^c^	22 ± 1.7^a^^,^^b^^,^^c^	16 ± 0.9^a^^,^^b^^,^^c^	25.8 ± 0.9^a^^,^^b^^,^^c^^,^^d^^,^^e^

Values are expressed as Mean ± SD for ten rats in each group and were considered significantly different at *p* < 0.05.

a Significantly different when compared to control group.

b Significantly different when compared to group Control + *C. aronia*.

c Significantly different when compared to group HFD.

d Significantly different when compared to group HFD + *C. aronia*.

e Significantly different when compared to the group HFD + SIM. HFD: high-fat diet; SIM: simvastatin.

f Significantly different when compared to group HFD then *C. aronia*.

### Changes in oxidative aorta stress parameters

**Figure 3.**
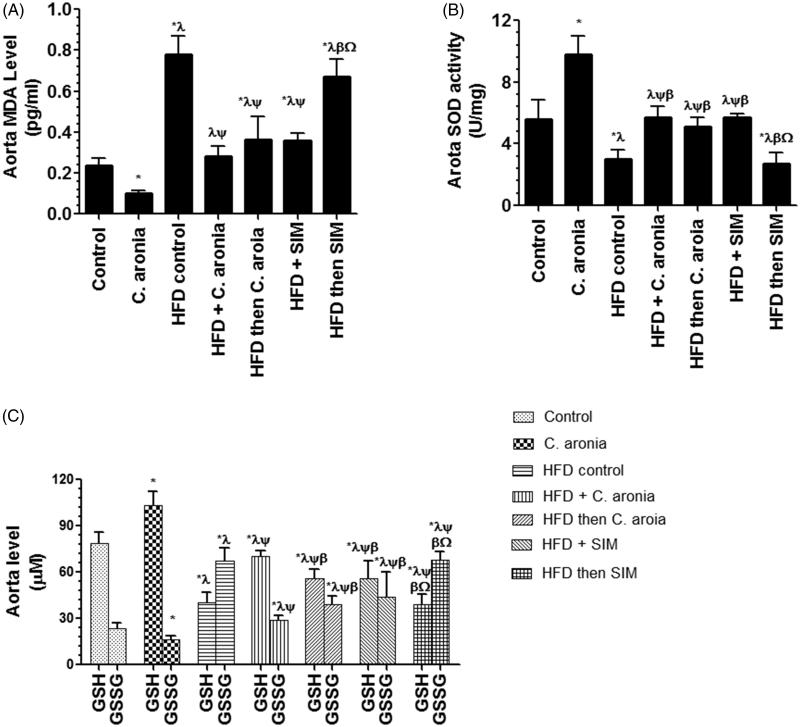
Levels of malondialdehyde (MDA, A), reduced glutathione (GSH, C), glutathione disulfide (GSSG, C) and activities of superoxide dismutase (SOD, B) in the aorta of all experimental groups. Values are expressed as Mean ± SD for ten rats in each group and were considered significantly different at P < 0.05. *Significantly different when compared to control group. ^λ^Significantly different when compared to group Control + *C. aronia*. ^ψ^Significantly different when compared to group HFDI. ^β^Significantly different when compared to group HFD + *C. aronia*. ^γ^Significantly different when compared to group HFD then *C. aronia*. ^Ω^Significantly different when compared to the HFD + SIM. HFD: high-fat diet; SIM: simvastatin.

Control rats received *C. aronia* had a significant increase in the levels of GSH and activity of SOD and a significant decrease in the levels of MDA and GSSG in their aorta homogenates as compared to the control group fed STD only (Figure 3(A–C)). On the other hand, HFD-fed rats had a significant increase in the aorta levels of MDA and GSSG and a significant decrease in the levels of GSH and activity of SOD as compared to their corresponding levels measured in the aorta homogenates of the control rats fed STD (Figure 2(A–C)). Similar findings were seen in the aorta of HFD-fed rats post-treated with SIM and were not statistically different as compared to HFD-fed rats. However, a significant decrease in the levels of MDA and GSSG with a concomitant increase in the levels of GSH and activity of SOD were seen in the aorta homogenates collected from of HFD-fed rats co-treated with SIM or *C. aronia* or post-treated with *C. aronia* as compared to HFD-fed rats received the vehicle (Figure 3(A–C)). Among these groups, the levels of MDA, GSH and GSSG and activity of SOD were not statistically different between the control rats fed STD and HFD+ *C. aronia-*treated rats.

### Changes in serum levels of inflammatory markers and adhesion molecules

**Figure 4.**
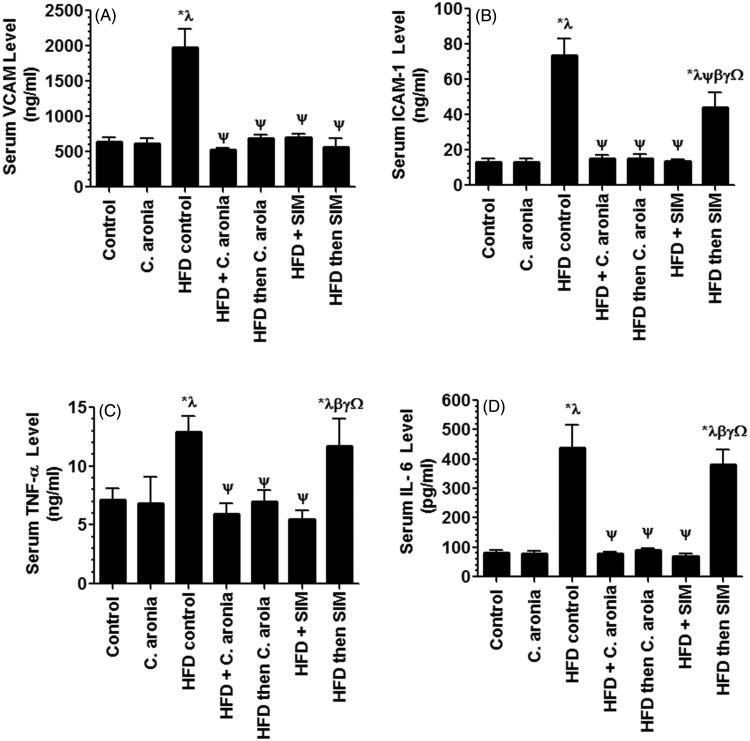
Levels of intracellular cell adhesion molecule (ICAM, A) and vascular cell adhesion molecule (VCAM, B), tumor necrosis factor (TNF-α, C) and interleukin-6 (IL-6, D)) in the serum of the control and experimental groups of rats. Values are expressed as Mean ± SD for ten rats in each group and were considered significantly different at *p* < 0.05. *Significantly different when compared to control group. ^λ^Significantly different when compared to group Control + *C. aronia*. ^ψ^Significantly different when compared to group HFDI. ^β^Significantly different when compared to group HFD + *C. aronia*. ^γ^Significantly different when compared to group HFD then *C. aronia*. ^Ω^Significantly different when compared to HFD + SIM. HFD: high-fat diet; SIM: simvastatin.

Serum levels of hsCRP (Figure 2(B)), ICAM-1, VCAM, TNF-α and IL-6 (Figure 4(A–D)) were not significantly different between the control rats received the vehicle of *C. aronia*. Serum levels of all these inflammatory markers were significantly increased in the group of rats fed HFD (Figures 2(B) and 4(A–D)). Similarly, serum levels of hsCRP, ICAM-1, TNF-α and IL-6 were also significantly increased in the serum of HFD then SIM-treated rats and their levels were not significantly different as compared to HFD-fed rats (Figures 2(B) and 4(B–D)). Levels of VCAM were the only parameters decreased in the serum of HFD then SIM-treated rats (Figure 4(A)). However, a significant decrease in the levels of hsCRP, ICAM-1, VCAM, TNF-α and IL-6 was seen in the sera of rats received in addition to HFD, concomitant doses of *C. aronia*, SIM or post-treated with *C. aronia*, with no significant differences in the levels of these markers between the same groups, and the control rats fed STD (Figures 2(B) and 4(A–D)).

### Changes in the aorta protein expression of TNF-α and IL-6

**Figure 5.**
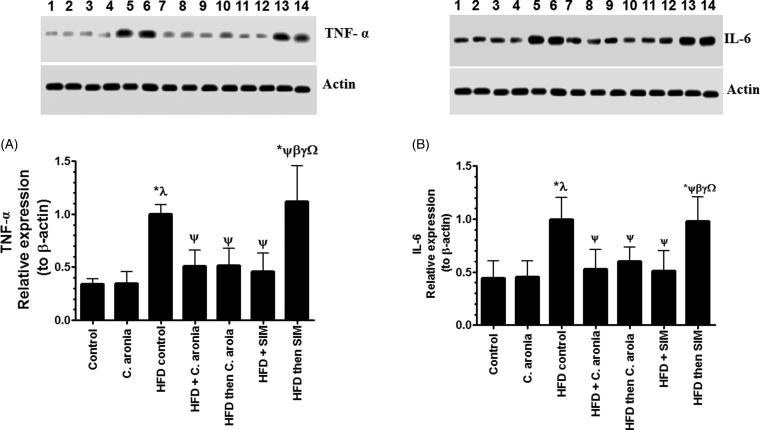
Protein levels of TNF-α (A) and IL-6 (B) in the aorta of all experimental groups as normalized to the relative expression of β-actin. Values are expressed as Mean ± SD for six rats in each group and were considered significantly different at *P* < 0.05. *Significantly different when compared to control group. ^λ^Significantly different when compared to group Control + *C. aronia*. ^ψ^Significantly different when compared to group HFDI. ^β^Significantly different when compared to group HFD + *C. aronia*. ^γ^Significantly different when compared to group HFD then *C. aronia*. ^Ω^Significantly different when compared to HFD + SIM. HFD: high-fat diet; SIM: simvastatin.

Protein levels TNF-α and IL-6 were abundantly expressed in the aorta of all groups of rats and were within the expected size (Figure 5(A,B)). Protein levels of TNF-α and IL-6 were not significantly different in the aorta of control rats treated with *C. aronia* but were significantly increased in the aorta of HFD-fed rats received the vehicle or post-treated with SIM (Figure 5(A,B)). Protein levels of TNF-α and IL-6 were not significantly different when HFD and HFD then SIM were compared with each other (Figure 5(A,B)). On the other hand, levels of both TNF-α and IL-6 were significantly decreased in the aorta of HFD-fed rats which received concomitant doses of *C. aronia*, SIM or post-treated with *C. aronia* as compared to HFD-fed rats (Figure 5(A,B)). Interestingly, there were no significant differences in the expression of TNF-α and IL-6 between the groups of HFD-fed rats *C. aronia* or SIM or post-treated with *C. aronia* and the levels of these markers were not statistically different with their corresponding levels detected in the control group fed STD (Figure 5(A,B)).

### Histology changes of the aorta and histometric analysis

**Figure 6.**
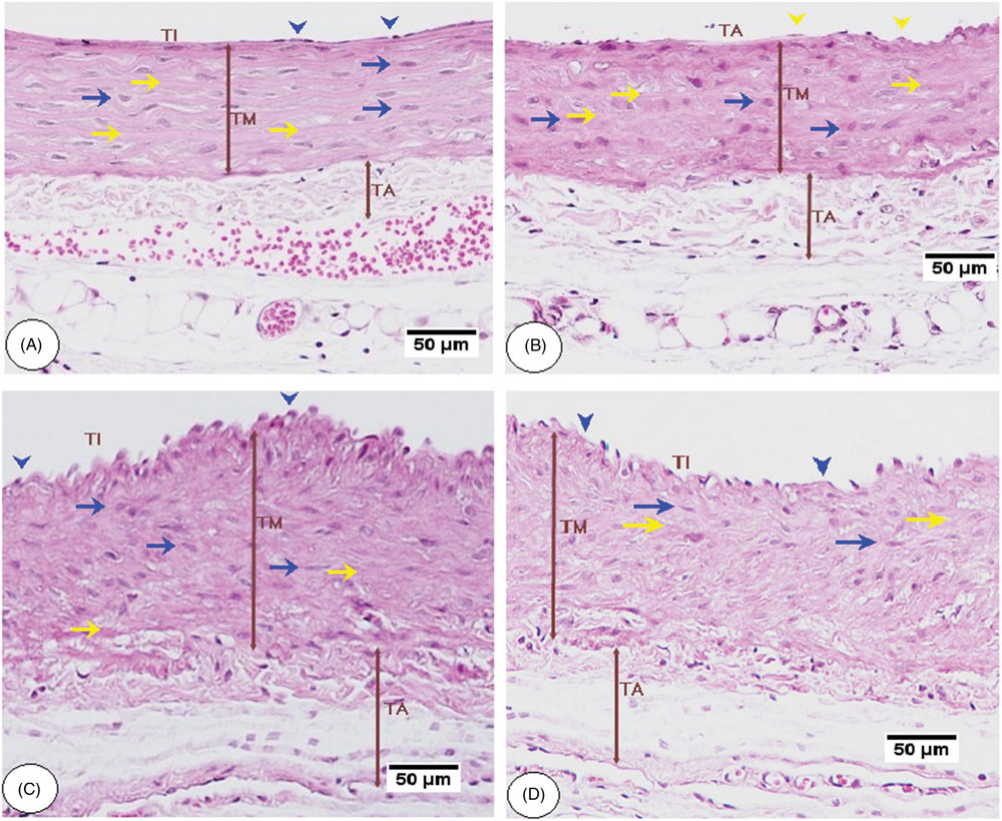
Aorta histopathology of control rats fed a standard (STD) and high-fat diet (HFD) rats. A and B were taken from a control rat fed STD, and a control rat fed STD and co-treated with *C. aronia*, respectively. All layers including tunic intima (TI), media (TM), and adventitia (TA) were visible with normal diameter and appearance. Endothelial cells are visible in the surface of the vessel lumen (blow arrowheads), and the medial SMCs of the tunica media are oriented horizontally to the aortic canal (blue arrow). Abundant and well-formed elastic fibres were displayed in a lamellar pattern (yellow arrow), and the TA shows few elastic fibres in the form of a loose network. C and D were taken from HFD-fed rats, and show showed disturbance and proliferation of tunica TI layer, the disorientation of SMCs with unclear borders and loss of elastic lamellae architecture. They also show the markedly increased thickness of TM and TA. H&E, 200×

**Figure 7.**
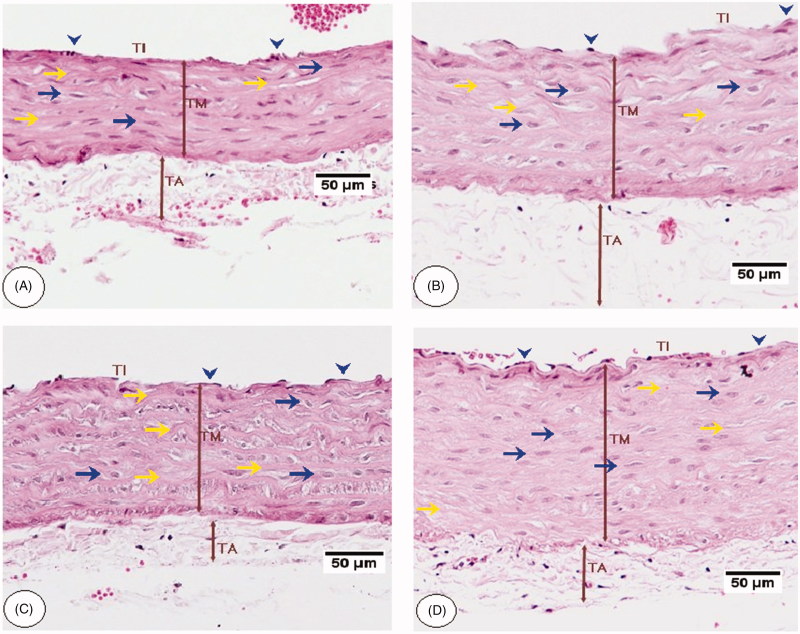
Aorta histopathology of high-fat diet (HFD) treated rats. (A) was taken from an HFD diet-fed rat co-treated with *C. aronia* (STD + C. aronia). (B) was taken from an HFD-fed rat post-treated with *C. aronia*. (C) was taken from an HFD-fed rat post-treated with SIM. These sections showed intact endothelial cells lining the tunica intima (TI) (blue arrowheads), horizontally oriented SMCs of tunica media (TM) toward the aortic canal (blue arrows). Also, well-formed elastic fibres were displayed in a lamellar pattern in its TM (yellow arrows) with marked decreases in the thickness of TM and TA. (D) was taken from an HFD-fed rat post-treated with SIM and shows similar findings like those observed in HFD-fed rats including disturbance and proliferation of tunica TI layer, the disorientation of SMCs with unclear borders and loss of elastic lamellae architecture. H&E, 200×.

**Figure 8.**
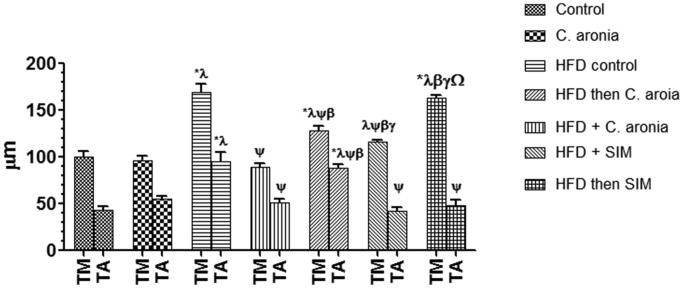
Histometry analysis of the aorta layers thickness in all experimental groups. Values are expressed as Mean ± SD for ten rats in each group and were considered significantly different at *p* < 0.05. *Significantly different when compared to control group. ^λ^Significantly different when compared to group Control + *C. aronia*. ^ψ^Significantly different when compared to group HFDI. ^β^Significantly different when compared to group HFD + *C. aronia*. ^γ^Significantly different when compared to group HFD then *C. aronia*. ^Ω^Significantly different when compared to HFD + SIM. HFD: high-fat diet; SIM: simvastatin.

Histological analysis by H&E staining of the aorta of the control rats fed STD or STD-fed rats co-treated with *C. aronia* showed intact tunica intima with simple squamous endothelial lining. Most of the SMCs of the tunica media are oriented horizontally to the aortic canal. Abundant and well-formed elastic fibres were displayed in a lamellar pattern, but the tunica adventitia shows few elastic fibres in the form of loose network unlike the lamellar arrangement in the media (Figure 6(A,B)). HFD-fed rats (Figure 6(C,D)) or HFD rats post-treated with SIM (Figure 7(D)), showed disturbance and proliferation of tunica intima layer, the disorientation of SMCs with unclear borders, loss of elastic lamellae architecture and markedly increased the thickness of tunica media and adventitia (Figure 8). HFD-fed rats co-administered *C. aronia* (Figure 7(A)), SIM (Figure 6(C)) or post-treated with *C. aronia* (Figure 7(C)) showed less remarkable abnormalities; the ECs lining of the tunica intima are intact, and most of the SMCs of the tunica media are oriented horizontally to the aortic canal. Also, the aorta sections of all these groups show well-formed elastic fibres that are oriented in a lamellar pattern in their tunica media. The thickness of the tunica media and adventitia are significantly decreased in all treated groups with profound reduction observed in the group of rats received the concomitant dose of *C. aronia* (Figure 8).

## Discussion

The HFD regimen used in this study induced persistent hyperlipidemia raised circulatory levels of ox-LDL-c and consequently induced an inflammatory response. The levels of tested inflammatory markers including TNF-α, IL-6 and hsCRP were significantly increased in the serum of HFD model rats and associated with a parallel increase in the expression levels of TNF-α, IL-6, enhanced aortic oxidative stress and increased intima-media thickness (IMT) in their aortas. These changes occurred despite stopping the HFD for 4 weeks. However, the co-administration and post-administration of *C. aronia* and co-administration of SIM significantly ameliorated the inflammatory marker levels and reduced the aorta IMT with more profound changes seen when *C. aronia* is administered concomitantly with HFD.

The first observation in this study is the significant decrease in the levels of TGs, CHOL, and LDL-c in the sera of STD-fed rats administered *C. aronia* and HFD-fed rats co-administered or post-treated with *C. aronia*. In the same line with other authors (Al-Hallaq et al. 2012), we have previously reported the similar hypolipidemic effect of *C. aronia* in both control and HFD-fed rats (Humayed 2017). Similar mirror image picture was obtained in the sera of HFD-fed rats co-administered SIM but not in the sera of rats post-treated with SIM. This is expected as SIM is an inhibitor of 3-hydroxy 3-methyl glutaryl COA reductase (HMG-CoA reductase) that results in decrease in CHOL synthesis and increase in LDL receptors expression (more LDL clearance), an effect that has been shown to be highly active during the development of hyperlipidemia and less effective after its development (Bach et al. 1990).

Numerous studies have investigated the possible mechanisms behind the hypolipidemic effect of *C. aronia*. In this regard, it was shown that *C. aronia* could inhibit the intestinal acyl-CoA cholesterol acyltransferase (ACAT) activity but not the hepatic HMG CoA reductase or cholesterol 7α-hydroxylase (C-hx) activity (Khalil et al. 2008). *C. aronia* contains high content of dietary fibres (Zcan et al. 2005) which are known to reduce circulatory levels of LDL-c by inhibiting the absorption of bile acids and CHOL, enhancing the expression and activity of LDL-c receptors (Sedigheh et al. 2011), and reducing TGs levels by suppressing lipogenesis in the rat’s liver (Romero et al. 2002; Lecumberri et al. 2007). Furthermore, *C. aronia* aqueous extract is very rich in flavonoids such as vitexin-20″ *O*-rhamnoside, acetylvitexin-20″ *O*-rhamnoside, quercetin, and chlorogenic acid, all of which are well-characterized by their potent hypolipidemic effects and their ability to increase the number of LDL receptors on the surface of liver cells and reduce TGs synthesis (Chang et al. 2005; Meng et al. 2013). These studies could explain the decreases in levels of TGs, CHOL and LDL-c in the sera of both STD and HFD-fed rats co-or post-treated *with C. aronia.*

However, persistent hyperlipidemia can provoke ROS and generate peroxynitrite (OONO–) which ultimately lead to tissue oxidative stress by increasing the activities of three primary oxidant producing enzyme systems; NADPH oxidases, xanthine oxidase (XO), and myeloperoxidase (Cai and Harrison 2000; Balkan et al. 2002; Forstermann and Munzel 2006; Stokes et al. 2006; Schulz and Munzel 2008; Cave 2009). Free oxygen radicals can oxidize LDL-c leading to overproduction of ox-LDL-c, which can accumulate in the endothelium and is considered as a reliable marker of oxidative stress and atherogenicity (Balkan et al. 2002). This could explain the significant increases in serum levels of ox-LDL-c and the parallel rise in aorta homogenates levels of lipid peroxidation (MDA levels) and the significant decreases in GSH levels and SOD activity in the aorta homogenates of HFD-fed rats.

In this study, the reduction in the serum levels of LDL-c and ox-LDL-c observed with administration of *C. aronia* to STD-fed rats is associated with significant increase in the antioxidant potential of the aortic cells as noticed by the significant decreases in basal MDA levels and the significant increases in basal GSH levels and SOD activity. Similar effects are observed in HFD-fed rats co-or post-treated with *C. aronia* or co-treated with SIM. These findings strongly demonstrate the antioxidant potential of *C. aronia* in both control and HFD-fed rats and confirm the previous findings that SIM can also act as an antioxidant when administered as co-therapy (Li et al. 2010; Nikolic et al. 2018). Indeed, SIM was shown recently to increase the activities of SOD and catalase and levels GSH in numerous tissues of various animal models, independently of modulating eNOS or iNOS (Li et al. 2010; Spindler et al. 2012; Melo et al. 2013; Crespo and Quidgley 2015). Similar to our findings in the control rats fed STD, *C. aronia* crude extract has a potent ROS scavenging ability and can increase GSH levels *in vivo* in control rats (Ljubuncic et al. 2005). In this context, further research on the effect of *C. aronia* or SIM on the aforementioned ROS generation enzymes and their direct stimulatory effect of GSH and SOD expression need further investigation.

Interestingly, the HDL-c level is decreased in the sera of HFD-fed rats and significantly increased in the sera of the rats that were co-treated with *C. aronia* or SIM and post-treated with *C. aronia*. These findings could explain the association between HFD and increased ox-LDL-c levels. Indeed, HDL-c can prevent LDL-c oxidation by its direct antioxidant potential and prevents lipid hydroperoxides accumulation in LDL (Mackness et al. 1991; Durrington et al. 2001). Given the stable level of HDL-c in the sera of the control rats that were treated with *C. aronia*, the reduction in ox-LDL-c levels in the sera of these rats could mainly be attributed to the ability of *C. aronia* to reduce the LDL-c level and to its antioxidant potential rather than modulation levels of HDL-c. Moreover, the significant increase in HDL-c levels on the sera of rats co-treated with *C. aronia*, SIM or post-treated with SIM may also participate in their inhibitory effects on circulatory ox-LDL-c levels.

Ox-LDL-c was shown to stimulate the vascular inflammatory response by induction of proinflammatory cytokines and adhesive molecules in various animals (Cushing et al. 1990; Frostegård et al. 1992; Rosklint et al. 2002). Soluble forms of the adhesion molecules such as VCAM-1 and ICAM-1 can be detected in plasma and are a good indicator of their availability on the cell membrane (Raffray et al. 2017). Also, in preclinical practice and experimental studies, one surrogate marker to detect atherosclerosis is the measurement of blood vessel IMT (Stein et al. 2008). In the general population, the Offspring Cohort of the Framingham Heart Study (OCFHS) demonstrated a direct association between inflammatory markers and IMT (Stein et al. 2008).

In this study, the increased aorta IMT of HFD-fed rats indicate the proinflammatory action of HFD and is confirmed by the significant elevation in serum levels of the tested inflammatory markers including IL-6, TNF-α and hsCRP, adhesion molecules including ICAM-1 and VCAM-1 together with the significant increase in the aorta protein expression of both IL-6 and TNF-α. IL-6 which is mainly secreted by SMCs is an atherogenic cytokine that enhances the synthesis and release of atherothrombotic molecules such as fibrinogen and plasminogen activator inhibitor-1. It also stimulates the expression of chemokines and adhesion molecules (ICAM and VCAM) on the surface of the ECs and stimulates the synthesis and release of CRP from both hepatocytes and aortic ECs (Loppnow and Libby 1990; Zheng et al. 1995; Klouche et al. 2000; Von der Thusen et al. 2003; Bassuk et al. 2004; Girn et al. 2007; Thakore et al. 2007). CRP by itself is a pro-atherogenic and prothrombotic factor that promotes ECs dysfunction and monocyte-ECs adhesion and transmigration (Devaraj et al. 2005, 2009; Verma et al. 2006). CRP also increases tissue factor release and release of ROS and promotes ox-LDL-c uptake and could participate in the oxidative stress response in the aorta of rats fed HFD (Devaraj et al. 2005).

Similarly, TNF-α is involved in the atherosclerotic progression from the initial stages of IMT to the subsequent vessel occlusion (Barath et al. 1990). It stimulates E-selectin and adhesion molecule expression by ECs, SMCs, and macrophages, promoting interaction between endothelium, leucocytes, and platelets, thus creating a pro-inflammatory and prothrombotic local environment (Galis et al. 1995; Young et al. 2002). Also, previous reports show that TNF-α induces IL-6 in many types of cells (Sawada et al. 1992; Hashizume et al. 2008). The increase in the serum and aorta levels of TNF-α post HFD feeding may explain the subsequent origin of the rise in serum and aortic levels of IL-6 and hsCRP which could explain the source of the inflammatory response after HFD feeding.

What is considered unique in this study is the amelioration of all these inflammatory markers in HFD-fed rats after co- or post-administration of *C. aronia* or post-administration of SIM. Similar to our findings, statin has been reported previously to inhibit CRP reduction by modulation of inflammatory cytokines action, and the mechanism remains unclear (Voleti and Agrawal 2006). From the first glance, this could be attributed to the decreased levels of ox-LDL-c in the sera of the treated rats leading to a decrease in the inflammatory markers levels. However, given the stable serum levels of ICAM-1, VCAM, IL-6, TNF-α, and hsCRP and the expression levels of IL-6 and TNF-α in aorta of the control STD-fed rats received *C. aronia*, despite the decrease in ox-LDL levels, we could conclude that the inhibitory action of *C. aronia* on the expression of these inflammatory markers is more active during HFD-fed condition. This could be mediated by another upstream mechanism that is activated under HFD-fed conditions. However, further investigation is needed in this regard.

## Conclusions

Overall, the present study indicates the beneficial effect of *C. aronia* in preventing and treating hyperlipidemia, oxidative stress and vascular inflammation. The possible mechanism evading vascular dysfunction may not be limited to the effect of the extract on gene expression of the tested inflammatory cytokines and molecules but predominantly involves the antioxidant potential through ameliorating the altered serum levels of ox-LDL and HDL-C.
